# A database of hourly wind speed and modeled generation for US wind plants based on three meteorological models

**DOI:** 10.1038/s41597-023-02804-w

**Published:** 2023-12-08

**Authors:** Dev Millstein, Seongeun Jeong, Amos Ancell, Ryan Wiser

**Affiliations:** Energy Analysis and Environmental Impacts Division, Lawrence Berkeley National Laboratory, Berkeley, CA 94720 USA

**Keywords:** Atmospheric science, Energy supply and demand, Climate and Earth system modelling, Wind energy

## Abstract

In 2022, wind generation accounted for ~10% of total electricity generation in the United States. As wind energy accounts for a greater portion of total energy, understanding geographic and temporal variation in wind generation is key to many planning, operational, and research questions. However, *in-situ* observations of wind speed are expensive to make and rarely shared publicly. Meteorological models are commonly used to estimate wind speeds, but vary in quality and are often challenging to access and interpret. The Plant-Level US multi-model WIND and generation (PLUSWIND) data repository helps to address these challenges. PLUSWIND provides wind speeds and estimated generation on an hourly basis at almost all wind plants across the contiguous United States from 2018–2021. The repository contains wind speeds and generation based on three different meteorological models: ERA5, MERRA2, and HRRR. Data are publicly accessible in simple csv files. Modeled generation is compared to regional and plant records, which highlights model biases and errors and how they differ by model, across regions, and across time frames.

## Background & Summary

The hour-to-hour profile of wind speed at wind turbines and the resulting profile of generation is critical input for a wide range of applications. For example, the match between hourly wind generation and hourly electricity demand can impact assessments of the value of wind plants^1–6^, the timing of wind output can influence operational decisions across power grids^7,8^, and can even impact long term planning^9–12^. With respect to decarbonization goals, Jenkins *et al*.^13^ review decarbonization modeling studies and find that important “…challenges associated with the variability of wind and solar increase nonlinearly as the share of energy from these sources rises.” Detailed understanding of hourly wind speed profiles is also needed to evaluate new wind plant technologies and system controls strategies, such as systems that minimize wake losses^14–16^, or turbine configuration options designed to maximize output at different wind speeds^17^.

While the importance of understanding variation in hourly wind speeds and generation is clear, there is a dearth of publicly available observational records that can be used to assess wind speed patterns at the relevant heights above ground and close to actual wind plants^18^. For example, Ramon *et al*.^19^ compile a global data set of tall tower measurements showing sparse coverage in wind energy regions in the United States. Though wind generation estimates are sensitive to many inputs, including plant layout, turbine characteristics, turbulence, and wind direction, Monforti and Gonzalez-Aparicio^20^ demonstrate the relative importance of wind speed fields as critical to accurate modeling of wind generation profiles.

One challenge to obtaining the needed observations is that measuring wind speeds at heights relevant to wind generation is expensive, requiring tall towers or remote sensors. The average tip height (from ground to the top of a vertically positioned blade) for new wind plants in the United States in 2021 was 158 meters above ground, and the center of the swept area of a wind turbine (often called the ‘tower height’ or ‘hub height’) was 94 meters above ground^6^. As the tip heights of wind turbines have increased to above ~100 meters, approximating hub-height wind speeds based primarily on ground-level measurements is no longer applicable^18,21–24^, though sounding data can be used to provide information about the vertical profile of wind speed and used in combination with surface measurements^25,26^. A further challenge is that private entities (often wind plant developers or operators) that do measure wind speeds at hub heights tend to keep their data private to maintain a competitive advantage versus competitors^27^.

Due to the above-mentioned challenges, the wind energy research community commonly depends on meteorological models for wind data, for example see refs. ^28–30^. Meteorological models provide geographically and temporally complete coverage of wind speeds. We discuss two types of meteorological models. The first type is reanalysis, which usually provides global coverage of historical meteorology using a fixed set of methods over time. The second type is operational forecasting, which provides high-resolution forecasts of near-term and real-time meteorology, often focusing on selected regional areas rather than the whole globe. Both types of meteorological models are constrained by meteorological observational data sets, including from surface monitors and satellites. We focus on two publicly available global reanalysis products, ERA5 (European Centre for Medium-Range Weather Forecasts Reanalysis version 5)^31^ and MERRA2 (Modern-Era Retrospective analysis for Research and Applications, Version 2)^32^, and one regional forecasting model, HRRR (High-Resolution Rapid Refresh)^33^. We note that these meteorological models are commonly used in the wind research community.

Meteorological models display structural biases and errors. Several research efforts assess meteorological models in regard to their use in wind energy applications. For example, Gualtieri^34^ evaluated ERA5 wind speeds at six tall towers around the world, finding substantial biases at towers near complex terrain. Staffell and Pfenninger^35^ find strong and varying spatial bias in modeled wind capacity factors based on MERRA and MERRA2 across countries in Europe. Jourdier^36^ assess the performance of ERA5, MERRA2, and other more highly resolved meteorological models in France, finding that accuracy and biases vary substantially by model and terrain type. Murcia *et al*.^37^ find a similar variety in performance when evaluating ERA5 and other regional meteorological models across countries in Europe.

Some efforts have also focused on evaluating meteorological models in the United States. For example, Olauson^38^ examined a region in the northwest U.S. (as well as countries in Europe) and found ERA5 performed better than MERRA2 as a primary input to regional wind energy modeling. Coburn^39^ evaluated surface wind speeds in 6 global reanalysis models, finding that newer models generally performed more accurately but still maintained biases. Davidson and Millstein^40^ assess wind generation modeled using ERA5, MERRA2, and HRRR by comparing modeled wind generation to recorded generation at over 100 individual wind plants in Texas. Davidson and Millstein^40^ find that the higher-resolution model, HRRR, performs best, but that model errors increased at nighttime, possibly due to misrepresentation of the surface boundary layer. In the United States, the WIND Toolkit also provides highly resolved regional model output with data available from 2007–2013, and has been validated for use in wind integration studies^41,42^. In comparison to ERA5, the WIND Toolkit was found to have lower overall bias but over predict the amplitude of the wind speed diurnal cycle^43^.

Despite these efforts, investigation of meteorological model performance has been limited by the effort required to gather, process, clean, and interpret necessary data from many sources. Efforts to simplify the data gathering do exist; for example, Renewables Ninja^35,44^, an online tool available at www.renewables.ninja, allows users to quickly build hourly generation profiles for wind and solar plants anywhere in the world. For wind power, the tool is based primarily on MERRA2 wind speeds, and its authors describe how bias correction at the country level can improve modeled generation.

The Global Wind Atlas, www.globalwindatlas.info, provides high-resolution (250 m) wind resource mapping to help policy makers, planners, and developers identify and assess promising wind resources. For example, it can be combined with additional data on land use restrictions to provide regional assessments of wind resource^45^. The Global Wind Atlas uses a downscaling process, with large-scale wind and climate data provided by ERA5. The Global Wind Atlas provides statistics about temporal variation in wind resources at all locations but does not provide hourly time series estimates of wind speed or generation.

In addition to databases of wind speed and generation, several tools support the downloading and processing of meteorological data. One such tool is the Geodata python library^46^ (www.github.com/GeodataTools/geodata). The motivation for Geodata is to simplify the process of working with earth systems data and thus reduce ‘start-up’ barriers for researchers.

Despite efforts such as the above to provide wind speed and generation information to researchers, substantial effort is still required to model and then validate the output across a large fleet of wind plants. In Europe, additional resources are available that provide hourly data and modeled wind generation for onshore and/or offshore wind plants^47,48^. These data sets are not available in the U.S. The PLUSWIND data repository, developed by the authors and described herein, addresses this data gap by providing fully processed, easy to use, hourly wind speed and modeled generation data for most wind plants across the United States.

The PLUSWIND repository provides a unified set of hourly wind speed and generation estimates based on information from three meteorological models; from multiple sources of data about operational wind turbines (through the USWTDB, EIA); and from a power generation simulation model (SAM). Wind speed and generation estimates are provided at almost all wind plants in the contiguous United States for the years 2018–2021. The data in PLUSWIND is contained in a simple table format (.csv file) and requires no coding of any sort to access. To keep the database simple and accessible, we have not included wind direction (only speed and generation). We hope that the low barrier to access will facilitate analysis of modelled wind speed and generation estimates across topics, including topics related to the integration of wind power generation into the electricity grid and the comparison and evaluation of meteorological model wind speed representation within the context of wind energy applications.

To provide insight into the accuracy of the PLUSWIND generation estimates, we compare generation estimates to records of generation from individual plants and regional electric system operators (i.e., Independent System Operator, ISO, regions and Regional Transmission Organization, RTO, regions). These comparisons can be found in the *Technical Validation* section. The comparison to recorded generation explores how biases differ among meteorological models. We analyze biases across different time frames, including seasonal and daily cycles, and long-term biases. While these comparisons can provide basic intuition into where and when the different models may lack accuracy, the *Technical Validation* section is not meant to provide a formal evaluation of model skill, or to pick a ‘winning’ model. In fact, one purpose of PLUSWIND is to facilitate new efforts to understand the causes of differences between meteorological models, to analyze model skill, and to allow detailed comparisons across different geographic regions.

**Fig. 1:**
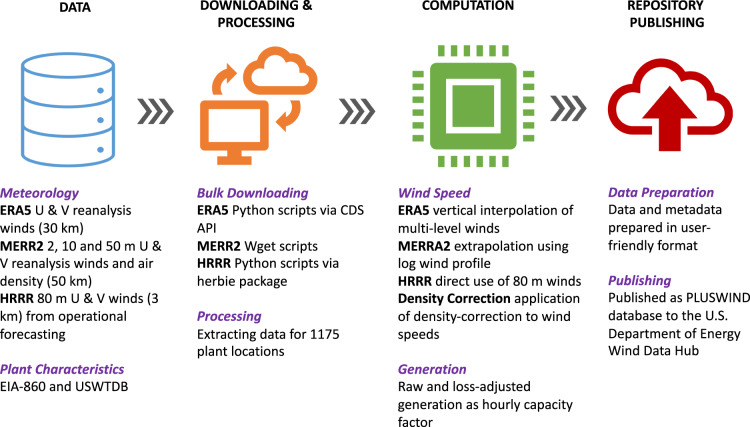
Flow diagram of process to create PLUSWIND data base.

Figure 1 provides a high-level summary of the approach used to develop PLUSWIND. Figure 1 highlights that somewhat different wind speed data are available from each model, and thus slightly different processes were used in each case to find wind speeds at the appropriate height above ground. A detailed description of the methodology is included in the following sections.

## Methods

### Wind plant characteristics

**Fig. 2:**
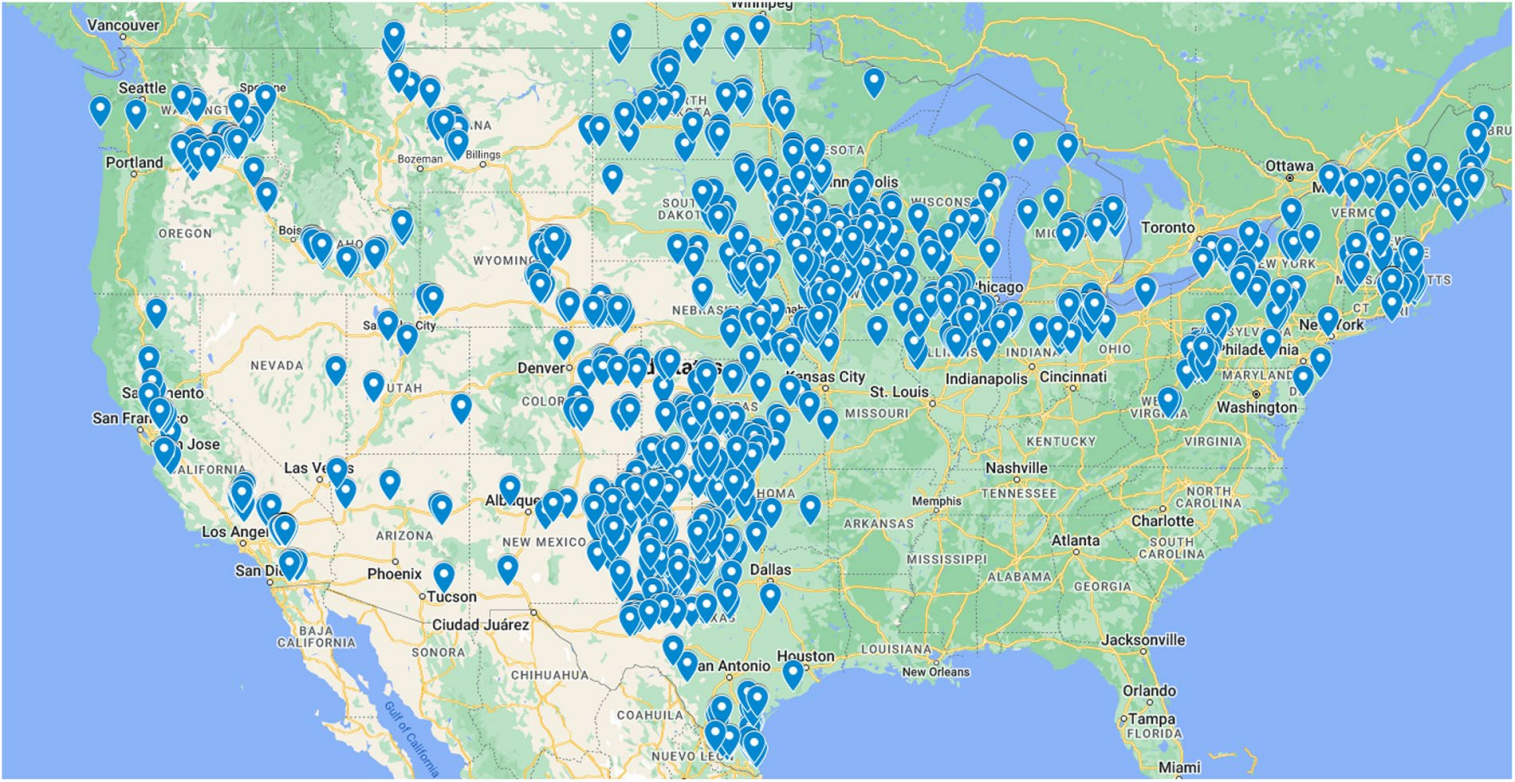
Map of all wind plants included in PLUSWIND. Shown is a screenshot of a Google map accompanying the PLUSWIND database in the Wind Data Hub.

We attempted to find wind speeds and generation estimates for all utility-scale (>1 MW) wind plants in the contiguous United States that were commissioned in or before 2020 and were operational during the study period (2018–2021). Figure 2 shows the location of all the wind plants included in PLUSWIND. In total we include 1175 wind plants. We used two sources of data to characterize each wind plant: (1) the United States Wind Turbine Data Base (USWTDB)^49^ and (2) U.S. Energy Information Administration Form 860 data^50^. To define plant centroids and hub height we used data from USWTDB, or if unavailable, we used data from EIA860. Note that there are sometimes small differences in location and hub-height information between the USWTDB and EIA860. We preferentially chose USWTDB characteristics in those situations. We excluded plants in cases when location or hub-height information was missing from both data sets. Plant-average specific power was calculated based on turbine diameter and capacity data within the USWTDB. A small number of mostly older plants (commissioned prior to 2008) were missing data to calculate specific power; in these cases, we used the average specific power from plants commissioned within plus or minus one year of the plant (e.g., if a plant commissioned in 2001 was missing turbine capacity and diameter data, we assumed its specific power equaled the average specific power across all plants commissioned in 2000, 2001, and 2002). In the Technical Validation section, we analyze the errors and biases of plants in the major wholesale electricity markets of the United States. Plants are associated with each market based on EIA860 data.

### Wind turbine power curves

**Fig. 3:**
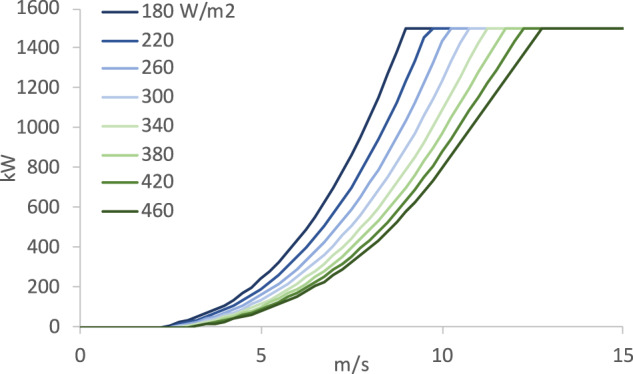
The ‘S’-shaped portion of a sample of power curves based on differing assumptions of specific power. The curve varies based only on specific power assumptions, and for the purpose of this figure is based on a turbine with capacity of 1500 kW; in PLUSWIND, however, only normalized outputs of power curves are provided.

Power curves provide rough estimates of generation from a wind turbine as a function of wind speed and turbine characteristics. Typical wind turbine power curves have several key features: a cut-in point (i.e., wind turbines generate no power below a certain wind speed, modeled at ~3 m s^−1^); a rated speed, above which the turbine operates at close to 100% capacity (rated speeds range from ~9 to ~13 m s^−1^); and a cut-out speed, when the turbine output drops to zero (modeled at 25 m s^−1^). Between the cut-in speed and the rated speed, power curves typically follow an ‘S’ shape, flattening to 100% of possible output at wind speeds above the rated speed. Average annual wind speeds typically fall somewhere on the curved portion of the power curve. This means the point at which the turbine reaches its rated speed is a key piece of information needed to understand how turbine output varies. The rated speed varies with turbine specific power: lower specific power turbines reach rated power at lower wind speeds, increasing output at lower wind speeds by sacrificing some output during higher wind speed hours. Figure 3 shows the ‘S’- shaped portion of a sampling of the power curves used in this work.

There are many limitations to the use of simple power curves to model energy generation as a function of hourly wind speed. Even when modeling a single turbine, a power curve provides only a rough estimate of hourly output, which will vary across shorter time periods and vary with conditions other than wind speed, such as wind veer and sheer, turbulence, and other local conditions. In multi-turbine wind plants, the use of a power curve does not account for losses that arise due to turbine wakes. Also, because we limit our wind speed estimates to a single time series for each plant and meteorological model, our estimate does not account for variation of wind speeds across turbines within each plant, which is important to consider given the non-linear nature of turbine power curves^51^. Further, some manufactures have power curves with characteristics that are slightly different from our basic description, such as mildly downward sloping curves above the rated speed.

The above notwithstanding, power curves do roughly represent generation response to different wind speeds and allow for representation of one of the most important technological developments in wind plant design: the shift over time toward lower-capacity turbines^6^. Therefore, despite the aforementioned limitations, we believe the use of a simple power curve modeling approach allows for valuable comparison of generation implied by meteorological model hub-height wind speeds and investigation of the associated implications for wind power generation modeling.

We match a power curve to each wind plant based on the average specific power of the wind plant. We developed power curves by varying specific power by 10 W m^−2^ across a range of 180 to 490 W m^−2^. We also included a limited number of power curves at higher specific power for a few older plants. Power curves were derived from the System Advisor Model v2020.11.29, with the following relevant turbine characteristics: shear coefficient of 0.14, maximum Cp of 0.45, maximum tip speed of 80 m s^−1^, maximum tip speed ratio of 8, drive train design of 3-stage planetary, and advanced design for both tower and blade. Specific power was selected by altering rotor diameter but keeping rated output constant. For the purpose of power curve creation, tower height was not considered.

We note that some plants were repowered during the study period, which could mean that new installed turbines had different characteristics (usually lower specific power) than the prior turbines. In these cases, we attempted to assign the plant the newest available turbine characteristics. In our comparisons to recorded generation, we excluded plants during the year in which they were repowered; however, we only assign each plant one power curve for the time period, which means that some plants have power curves that do not represent their turbines prior to repowering. The USWTDB contains information on when plants were retrofit.

### Meteorological data

Reanalysis and operational forecasting share similar underlying numerical models and data assimilation techniques. However, reanalysis modeling differs from operational forecasting modeling in that the data assimilation and the numerical models are fixed for a reanalysis period^52^. Thus, reanalysis products are based on the same data assimilation system and forecasting model throughout the reanalysis period. Reanalyses are usually produced at a lower horizontal resolution than operational weather forecasts. In fact, the horizontal resolution is one of the most important differences between the models, with resolution equaling ~50 km, ~30 km, and 3 km, for MERRA2, ERA5, and HRRR. Operational forecasting models are subject to frequent changes in their systems due to software bugs or biases in data. Thus, operational model outputs are not ideal for compiling a long time series to understand changes in climate and meteorology over time. Reanalysis products are typically available at the regional (e.g., North American Regional Reanalysis) and global (e.g., MERRA2) scales. Regional reanalyses use a higher-resolution weather forecasting model utilizing boundary conditions from a global reanalysis.

The model, product, and variable names for wind speed calculations are presented in Table 1.

**Table 1:** Product and variable names for wind speed calculations by the model.

Model	Product/Sub-model Name^§^	Variable name*
ERA5	reanalysis-era5-complete^†^ (ERA5 model level data)	U (U component of wind), V (V component of wind)
MERRA2	tavg1_2d_slv_Nx^‡^ (2d, 1-Hourly, Time-Averaged, Single-Level, Assimilation, Single-Level Diagnostics)	U2M (2-m U wind), V2M (2-m V wind), U10M (10-m U wind), V10M (10-m V wind), U50M (50-m U wind), V50M (50-m V wind), and DISPH (zero plane displacement height)
HRRR	Lambert Conformal 3 km Resolution)	u-component_of_wind_height_above_ground (u-component of wind @ Specified height level above ground)^¶^
		v-component_of_wind_height_above_ground (v-component of wind @ Specified height level above ground)

§The explicit product or submodel name is included in the parentheses.

*The long name for each variable is shown in parentheses.

^†^This is the product name used in the Python-based CDS API.

^‡^In the MERRA2 model, this product name is represented by “data collection.”

^¶^The specified height level is 80 m above ground. The abbreviated variable names for HRRR are UGRD and VGRD.

For air density, we use the AIRDENS variable available in MERRA2’s data collection “inst3_3d_gas_Nv,” whose long name is “3d, 3-Hourly, Instantaneous, Model-Level, Assimilation, Aerosol Mixing Ratio Analysis Increments.” We interpolate 3-hourly model-level data to calculate hub-height air density using a cubic spline method. The 3-hour hub height air density is then interpolated into hourly data using a linear approximation method.

We used the Climate Data Store (CDS) API (https://cds.climate.copernicus.eu/api-how-to), which is Python-based. ERA5 model-level data are not listed in the CDS catalog and cannot be downloaded via the CDS Web interface; it is only available through the CDS API service. Using a Python script, we downloaded ERA5 model-level data stored in the Meteorological Archival and Retrieval System (MARS) tape archive.

MERRA2 data were downloaded using a command line package “Wget,” which retrieves files using popular Internet protocols (e.g., HTTPS, FTP). Our work requires downloading data in bulk. Thus we first made a data file list to be used in the Wget script for file downloading.

We used the Herbie Python package (https://github.com/blaylockbk/Herbie) for downloading HRRR. The Herbie package^53^ allows for downloading data from big data cloud platforms such as Amazon Web Services and Google Cloud. In this work, we used Google Cloud (https://console.cloud.google.com/marketplace/details/noaa-public/hrrr?project=d-lab-intro). We use hourly ‘analysis’ outputs from HRRR, which correspond to a 0 hour forecast from each hourly model cycle runtime (00–23 h).

All these downloading methods depend on a few factors, including network speed and the queues on the data server. For example, downloading ERA5 data from the MARS tape archive can lead to significantly longer retrieval than the CDS Web interface (https://confluence.ecmwf.int/display/CKB/How+to+download+ERA5).

### Deriving wind speed at the average hub height for each wind plant

As detailed above, each meteorological model provides a slightly different set of wind speed outputs. Therefore, we must apply different methods to convert each model’s raw wind speed outputs to wind speeds that are representative of hub-height wind speeds. In each case, however, we choose a representative horizontal grid location (i.e., a single grid cell from each model) determined by the centroid point of each wind plant. We note that in some cases plant dimensions are larger than HRRR grid cells, however, for the sake of simplicity, we maintained a single representative point for these cases.

#### ERA5

We make use of ‘model level’ data, available as part of the ERA5 global atmospheric reanalysis dataset^54^. This dataset resolves the atmosphere across 137 vertical levels, ranging from the Earth’s surface (i.e., Level 137) up to an altitude of ~80 km. These data are stored in the Meteorological Archival and Retrieval System (MARS) tape archive, which is managed by the European Centre for Medium-Range Weather Forecasts (ECMWF). For our study, we downloaded data corresponding to the lower 10 levels from the MARS archive, covering vertical elevations from 10 m to ~300 m above the surface.

For the ERA5 model we simply apply a vertical interpolation to wind speed data from multiple model levels to find a single wind speed at the hub height of each plant. In this case we use a cubic spline interpolation method. As vertical model layers are finely resolved near hub-height (typically 20 to 40 meters of height) this interpolation likely adds little bias or error beyond that which is already embedded in the raw ERA5 wind speed output.

#### MERRA2

MERRA2 has more limited wind speed data available. Wind speed at heights of 2, 10, and 50 m above ground were used to fit a linear regression, which was then applied in a log wind profile to compute wind speeds at the hub height. The approach to extrapolate MERRA2 wind speeds to hub height largely follows methods described in Staffell and Green^55^. Staffell and Green^55^ discuss the choice between the power law and the log law for this purpose, choosing the log law approach because it provided robust results across a wide range of speeds and hub-heights, whereas the applicability of the power law, particularly due to the uncertainty associated with the Hellman exponent, may vary with site and height. 50 meters above ground is below the typical hub height of 90 meters, therefore the extrapolation, while necessary, likely adds some error and possibly bias.

#### HRRR

HRRR wind speeds are available directly at 80 m above ground. This height is close but slightly lower than the hub height of most plants (most plants in PLUSWIND have hub heights ranging from 76 to 90 meters). The hub height differences here have the potential to introduce a small bias. For example, Bolinger *et al*.^56^ find that increasing hub height by ~50 meters (from 88 to 140 meters) increases modeled annual plant output by ~23% (shifting the median capacity factor in their study from roughly 30% to 37% across a wide range of locations in the United States). However most wind plants in PLUSWIND have hub heights within 10 meters of the HRRR height, suggesting that any biases due to hub-height mismatch with HRRR will be *much* smaller than 23%. Therefore, we deemed that the added complexity of extrapolating 80 meter data to individual plant hub heights would negate the possible benefit to increased accuracy, and we use the raw output from HRRR of wind speeds at 80 meters above ground as our hub height wind speed for all plants. Finally, we note that HRRR provides additional wind speed data at other heights (e.g., 10 meters, and at selected model layers) but we did not make use this additional wind speed information in this work.

### Estimating generation based on hub-height wind speed

Hourly generation is provided as a capacity factor (CF), or a fraction of total possible output for the hour. To find total energy (MWh) output in a single hour, one would simply multiply the hour’s CF by the plant’s capacity (MW) and by 1 hour.

The PLUSWIND repository contains three different generation profiles for each plant and meteorological model input: (1) A ‘raw’ generation estimate based only on the hub-height wind speed, (2) a density adjusted generation profile, and (3) a density and loss adjusted profile.

It is important to note the limited purpose of including density. Air density adjustments are required because the simple power curves that we use to estimate generation assume standard air density. The correction for air density is most important for wind plants that are situated at high elevation, as air density at these plants is almost always lower than at plants close to sea level. Additionally, air density tends to vary by season. There are daily cycles in air density, but the daily changes to air density are smaller than changes due to elevation or season. Therefore, the primary purpose of adjusting for air density is to remove a source of bias due to low air density at high elevation and seasonal changes to air density. Given the limited purpose for air density corrections, we determined that it was sufficient to use a single source for air density for all models (we use air density from MERRA2).

Likewise, there is a limited purpose of including the loss adjustments. Losses are highly variable by plant and hour and are complex to model or estimate. Therefore the purpose of our loss adjustments is to reduce the long term bias that would be introduced by not accounting for losses. We do not attempt to produce realistic plant-level losses in any particular hour but do attempt to produce a realistic level of losses across the fleet of plants and an annual cycle. Including the loss adjustments (and air density adjustment) allows for a more apples-to-apples comparison with recorded generation, at least when analyzing a set of multiple plants across time.

To account for air density that is different from the standard air density we create a wind speed profile that is adjusted by an air-density correction factor. This ‘corrected’ wind speed profile does not represent physical wind speed, but is only useful when used as input to estimate power generation given the non-standard air density. We develop an air density correction factor (Eq. 1) following the approach described in the IEC 61400-12-1^57^:

1 $${\rm{WS}}={{\rm{WS}}}_{{\rm{RAW}}}\times {({\rm{\rho }}/{\rm{\rho }}{\rm{0}})}^{1/3}$$

where WS is the density adjusted wind speed, WS_RAW_ is the raw wind speed from each model, ρ is the air density (kg/m^3^) from MERRA2, and ρ_0_ is the reference air density, which is set to a typical air density at sea level of 1.225 kg/m^3^.

Regarding losses, Clifton *et al*. (2016) report estimates of plant-level losses. Specifically, Clifton *et al*. (2016) find that wake losses range from 0% to 10% of annual output, and turbine performance losses range from 1% to 3%. We thus take a rough center point from that range and apply a constant loss of 7%. Jacobson *et al*. also estimate a ~7% wake loss on average for wind in a decarbonized energy system^58^. However, at high enough wind speeds, a plant may be able to compensate for wake or performance losses, therefore, we taper loses as speed rises above each plant’s ‘rated speed’ (Eq. 2). Though sophisticated methods exist for estimating instantaneous losses at each plant, the effort would require at least wind direction and detailed spatial representation of plant layout. We instead apply a constant loss rate of 7% and use a simple ‘rule of thumb’ approach to taper loses as wind speeds approach or exceed the rated wind speed. The loss (L) is computed as a function of wind speed (WS) and rated speed (RS, the speed at which a turbine reaches its full capacity):

2 $$L=\left\{\begin{array}{cc}0.07 & {\rm{WS}} < {\rm{RS}}--0.5\\ 0.07-0.07\times \frac{({\rm{WS}}-{{\rm{RS}}}^{* })}{2.5} & {\rm{RS}}-0.5\le {\rm{WS}}\le {\rm{RS}}+2.0\\ 0 & {\rm{WS}} > {\rm{RS}}+2.0\end{array}\right.$$

where RS* is RS - 0.5.

We note that wind plants with few (or a single) turbine(s) have lower (or zero) wake losses, so a limitation of this approach is that it will overstate wake losses for those plants.

### Comparison to generation records

In order to compare modeled generation to recorded generation, we attempt to make the data streams as consistent as possible. Hourly regional generation records and plant generation records are available from the EIA (EIA923^59^ and EIA930^60^). However, the generation records are reported after curtailment. We therefore adjust generation records upwards by the amount of curtailment that occurred. Curtailment of wind plants is reported at the regional level, in some regions it is reported hourly (CAISO, SPP, and ERCOT), and in other regions is reported monthly (MISO, PJM, NYISO, and ISO-NE). We use hourly and plant-level curtailment estimates developed in the Wiser *et al*.^6^ to adjust reported generation for plant-to-plant comparisons as well as for the regional comparisons. The estimates of plant-level curtailment from regional totals, or the estimate of hourly curtailment based on monthly totals, are not perfect and introduce a small amount of error into these comparisons. However, total curtailment during the study period was low, well below 5% in most regions and years, and thus an imperfect record of curtailment should have a very minor impact on these comparisons.

Regional hourly generation records occasionally had missing data for individual hours, where generation was either not reported at all, or reported as zero despite high generation in neighboring hours and high modeled generation. Missing hourly records were replaced by the simple linear interpolation of generation recorded on either side of the missing data. There were no sequences of consecutive missing data longer than 4 hours, so interpolation was deemed sufficient. In a limited number of hours, reported regional hourly generation after it was adjusted for curtailment was anomalously large (e.g., larger than the total installed capacity in the region). Therefore, maximum hourly generation in each ISO and year was limited to its 99.5 percentile maximum hourly generation for that ISO and year (i.e., hours with generation larger than the 99.5 percentile value were set to the 99.5 percentile value).

Modeled generation is based on the wind speeds reported by the various meteorological models as described above. However, while ERA5 and HRRR report instantaneous wind speed values, MERRA2 reports average wind speed across an hour period. As we are comparing to reported hourly average generation we average modeled generation from ERA5 and HRRR to create hourly average generation estimates (Eq. 3) rather than instantaneous estimates.

3 $$Ge{n}_{hrBegAvg}\left(t\right)=\frac{Ge{n}_{instant}\left(t\right)+Ge{n}_{instant}\left(t+1\right)}{2}$$

The comparison to plant-level reported generation excluded some plants. First, for the purpose of consistency with the regional comparisons, only plants from the selected regions were included. Second, data for plants were only included for a particular year if the reported capacity factor of the plant fell between 20% and 70%, eliminating plants with unrealistic output or that faced maintenance or other issues unrelated to the profile of wind speeds. Plants without 12 full months of generation data reported in a particular year were excluded from that year of comparison. Reported generation data were not considered during the first 12 months of plant operation, to deal with phased construction and other ‘teething’ issues. Plants which were indicated to be undergoing repowering (as defined in the USWTDB) were excluded during the year in which the repowering occurred.

## Data Records

PLUSWIND is available at the U.S. Department of Energy Wind Data Hub^61^, at https://a2e.energy.gov/project/pluswind, or at 10.21947/1903602, and provided in comma-separated values (CSV) format. While registration is required for data access there is no requirement for submission of personal data as long as a valid email address is provided. The dataset contains the fields detailed below. The column names in the CSV file include units, when available, for example, with units of m/s for wind speed columns.

### gmt

date and time in Greenwich Mean Time with YYYYMMDDHH format.

### ERA5 wind speed (m/s)

ERA5 raw wind speed at hub height.

### MERRA2 wind speed (m/s)

MERRA2 raw wind speed at hub height.

### HRRR wind speed (m/s)

HRRR wind speed at 80 m.

### MERRA2 air density (kg/m^3^)

MERRA2 air density at hub height.

### ERA5 density-corrected wind speed (m/s)

ERA5 density-corrected wind speed at hub height.

### MERRA2 density-corrected wind speed (m/s)

MERRA2 density-corrected wind speed at hub height.

### HRRR density-corrected wind speed (m/s)

HRRR density-corrected wind speed at 80 m.

### ERA5 CF (raw)

Capacity factor calculated from ERA5 raw wind speed.

### ERA5 CF (density adjusted)

Density adjusted value of ERA5 raw capacity factor.

### ERA5 CF (density and loss adjusted)

Density and loss adjusted value of ERA5 raw capacity factor.

### MERRA2 CF (raw)

Capacity factor calculated from MERRA2 raw wind speed.

### MERRA2 CF (density adjusted)

Density adjusted value of MERRA2 raw capacity factor.

### MERRA2 CF (density and loss adjusted)

Density and loss adjusted value of MERRA2 raw capacity factor.

### HRRR CF (raw)

Capacity factor calculated from HRRR raw wind speed.

### HRRR CF (density adjusted)

Density adjusted value of HRRR raw capacity factor.

### HRRR CF (density and loss adjusted)

Density and loss adjusted value of HRRR raw capacity factor.

The auxiliary file for plant information (e.g., mean location of turbines for the plant) includes the following information.

### EIA_ID

plant ID from EIA.

### lon (degree)

average plant longitude.

### lat (degree)

average plant latitude.

### hub height (m)

average plant hub height.

## Technical Validation

To provide context for how well the modeled wind speeds represent actual wind speeds, we provide some basic comparisons to recorded data. Because measurements of wind speed at hub height are not widely available, we compare the modeled generation to recorded generation. Our analysis in this section is meant to provide basic insight on where and for which use-cases the modeled generation and underlying modeled wind speeds may be most useful. The comparisons to generation are *not* meant to precisely determine which model is ‘best’ through, for example, measurements of model skill. We do hope, however, that more detailed analysis along these lines may be facilitated by the publication of this data set.

**Fig. 4:**
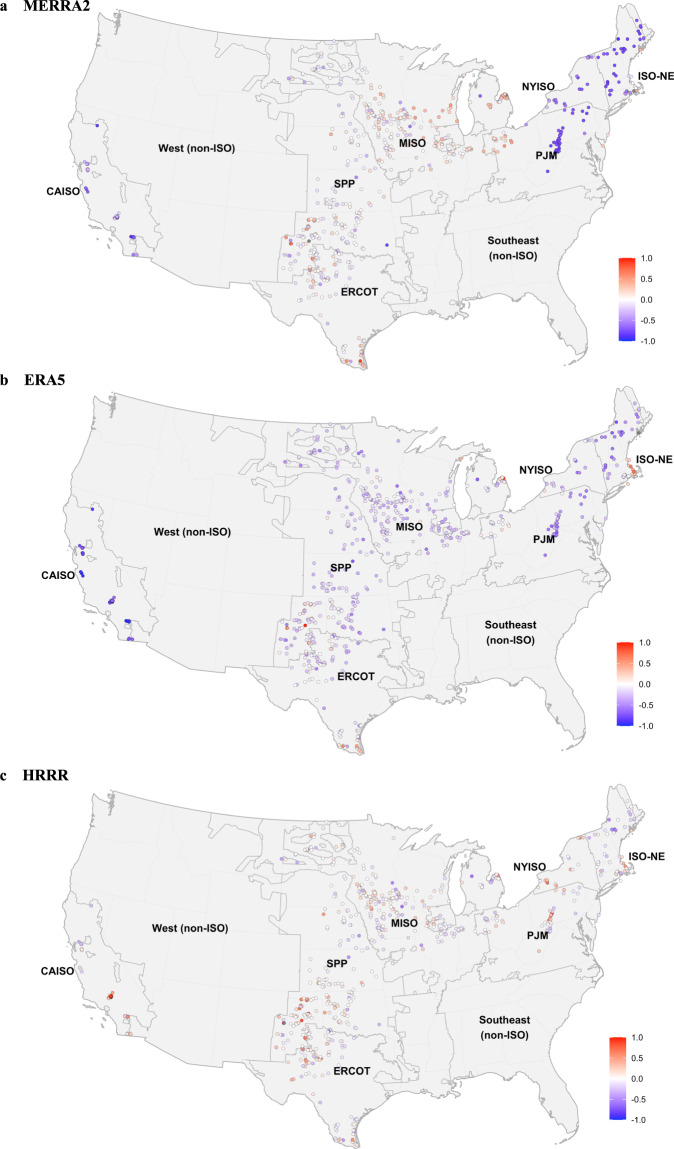
Mean normalized bias at each plant within the major wholesale electricity markets for the (**a**) MERRA2, (**b**) ERA5, and (**c**) HRRR models. Note that the PLUSWIND repository contains data for wind plants located in the “non-ISO West” region, but those were not evaluated versus recorded generation, as the evaluation is focused on the major wholesale market regions.

We analyze two types of wind generation data records: monthly generation reported by individual plants, and regional hourly generation reported across wholesale electricity markets. The seven wholesale markets we examine – CAISO, SPP, ERCOT, MISO, PJM, NYISO, and ISO-NE – are administered by Independent System Operators (ISOs) or Regional Transmission Organizations (RTOs) whose boundaries are shown in Fig. 4. Though PLUSWIND does contain data on wind plants located in the West (non-ISO) region, these plants are not evaluated here as we focus on plants within the major wholesale markets. To compare modeled generation with reported generation, the modeled generation must be summed across different dimensions. First, hourly modeled generation at each plant is summed across time to match the reported plant-level generation on a monthly basis. Second, hourly modeled generation is summed across all plants in a region to match regional hourly records. These comparisons provide insight into (1) long-term, plant-level biases and errors, and (2) the representation of hourly regional wind trends, including diurnal wind patterns. Additional details on the generation records can be found in the Methods section.

### Plant-level bias and error analysis

**Fig. 5:**
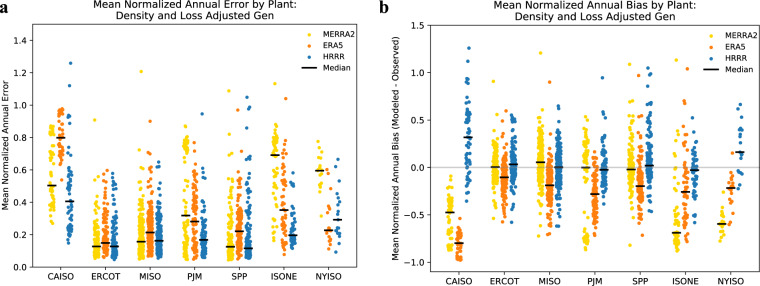
Error and bias varies by meteorological model, region, and plant. Mean normalized absolute error (**a**) and mean normalized bias (**b**) by plant. The normalizing mean is that of recorded generation, and the bias is modeled minus recorded generation. The black horizontal bar denotes the median value for each model-ISO combination.

The level of annual error and bias in PLUSWIND generation estimates varies substantially by region and model (Figs. 4, 5). For example, median normalized annual error (i.e., across all plants in a region) varies from 0.1 for HRRR-based generation estimates in SPP (centered in Kansas and Oklahoma) to 0.8 for ERA5-based generation estimates in CAISO (California). In other words, annual generation estimates based on ERA5 wind speeds are likely to have errors of ~80% relative to true values for plants in California, whereas estimates based on HRRR in SPP are likely to only have errors of ~10% compared to their true values. In the example above, we highlight a difference between region *and* meteorological model, but the level of error is sensitive to both individually. In the center of the country, SPP, MISO, ERCOT, and to a certain extent PJM errors are relatively low across all meteorological models, with most median errors falling between 10–20% of annual generation. In regions outside the center of the country, namely CAISO, ISO-NE, and NYISO, median errors typically fall between 30–80% of annual generation. Furthermore, these higher errors are mostly driven by systematic bias, with MERRA2 and ERA5 underestimating generation in these regions, and HRRR overestimating generation in these regions. Two exceptions to the above are that HRRR has relatively small errors and bias in ISO-NE, and ERA5 has relatively small bias and errors in NYISO.

One possible driver of the variation in errors and bias across regions is that the complex topography found in California, New York, and New England led to larger errors and biases compared to the regions in the center of the country. In California and New England, HRRR, which has finer resolution than ERA5 or MERRA2, tends to have lower errors and smaller biases. This raises questions for future research: does HRRR perform better in California and New England due to finer model resolution, or due to other differences in modeling methodology (e.g., HRRR assimilates radar data whereas MERRA2 and ERA5 do not)? How much improvement could be seen by explicitly modeling wake losses, or representing variation in wind speed across turbines (especially for plants located in complex topography)?

In addition to exploring median results in each region, we can also examine the spread of bias and error across plants. Errors and biases vary significantly across plants in each region (Figs. 4, 5). Errors at the individual plant can be caused by both errors in the meteorological modeling and by idiosyncratic issues related to the description of wind plants. For example, in some cases there are likely discrepancies between the reported capacity or turbine type and actual values. Additionally, our modeling does not account for extended maintenance requirements that might affect individual wind plants in some years.

### Regional diurnal generation

Hub-height wind speeds and generation tend to have distinct diurnal patterns that vary by region and season. These diurnal patterns are important for research related to wind energy grid integration and wind energy value because energy demand and prices also vary on seasonal and diurnal cycles.

We can evaluate model representation of hourly patterns across regions by making use of reported generation by the regional system operators. As mentioned earlier, this comparison requires the summation of generation across all individual plants in a region. One important limitation of this comparison is that most regions do not always report the exact set of plants included in their summation. We assume regions report all operating plants in their territory, but it is likely there are inconsistencies between the set of plants we use in each region and the set upon which reported totals are based. Therefore, in contrast to the preceding section, these comparisons do not provide a precise indication of overall bias. Rather, these comparisons provide general insight into how closely each meteorological model matches reported diurnal patterns as well as hour-to-hour variability.

**Fig. 6:**
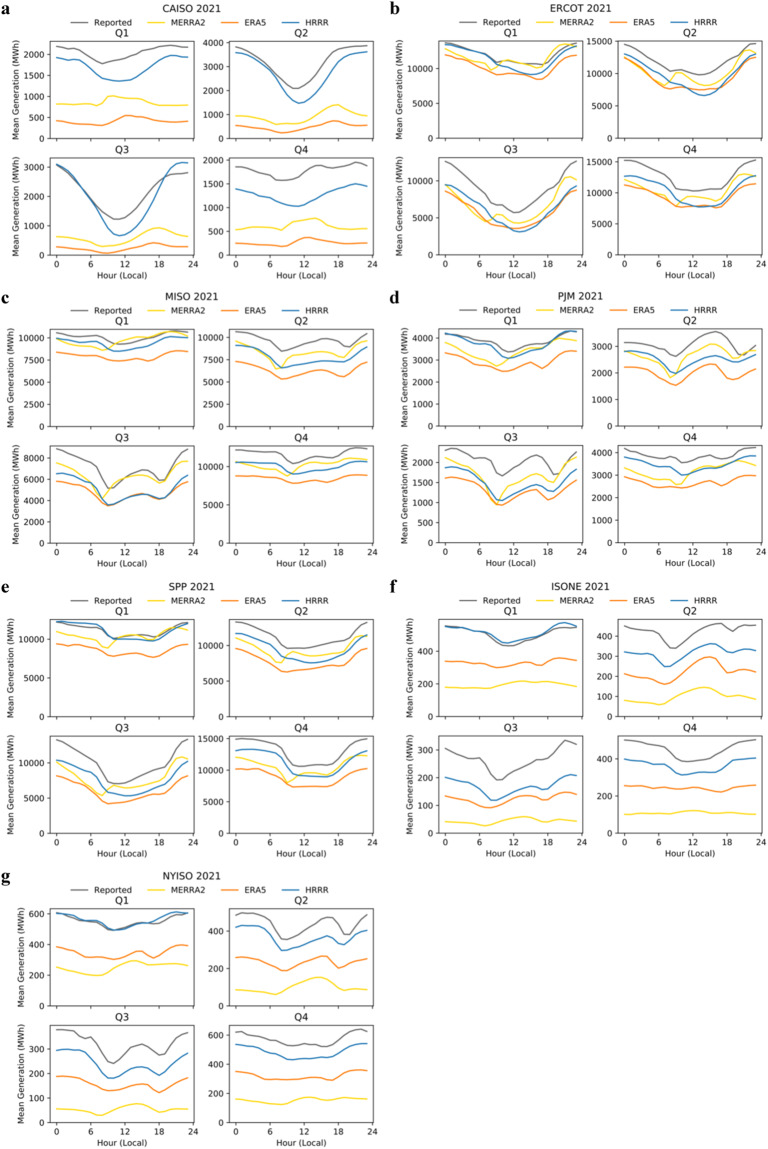
Seasonal and diurnal variation of recorded and modeled generation for 2021 by ISO. This figure should be used to compare diurnal cycle representation, but not overall bias (due to uncertainty in the set of plants included by each system operator report). Overall bias is best assessed in Figs. 4, 5. ISOs include (**a**) CAISO, (**b**) ERCOT, (**c**) MISO, (**d**) PJM, (**e**) SPP, (**f**) ISO-NE, and (**g**) NYISO.

In the four regions with relatively small annual model bias (i.e., SPP, MISO, ERCOT and PJM), visual inspection suggests that generation estimates based on HRRR and ERA5 roughly match the reported diurnal cycles (Fig. 6). By this we mean that while the lines for HRRR and ERA5 may appear below reported generation, the bias is roughly consistent across hours of the day. For example, HRRR and ERA5 correctly match the two minimum periods at 9:00 and 18:00 local time in Q3 in MISO. MERRA2 does not correctly match the diurnal cycle; generation based on MERRA2 is not evenly spaced from the recorded generation across hours of the day, in some cases varying from large under-predictions of generation to over-predictions of generation (Fig. 6). To provide a quantitative comparison, the coefficient of determination between the modeled and reported generation for the above example (comparing the 24 hourly average generation values in MISO, Q3), was 0.96 for HRRR and ERA5 but only 0.58 for MERRA2.

In the three regions with relatively large annual model bias (i.e., CAISO, NYISO, and ISO-NE), HRRR clearly captures more features of diurnal cycles than other models (Fig. 6). Most prominently, HRRR captures the strong midday minimum in CAISO in Q2 and Q3, whereas MERRA2 and ERA5 do not. In another example, HRRR captures the midday minimum in ISO-NE in Q4, while MERRA2 and ERA5 show relatively flat output across the day. In the preceding section, we indicated that finer model resolution was a plausible explanation for improvement in bias of HRRR; the same explanation is logical here for improvements in representation of the diurnal cycle, though further research would be useful to fully understand the reason(s) for improvements in HRRR representation.

**Fig. 7:**
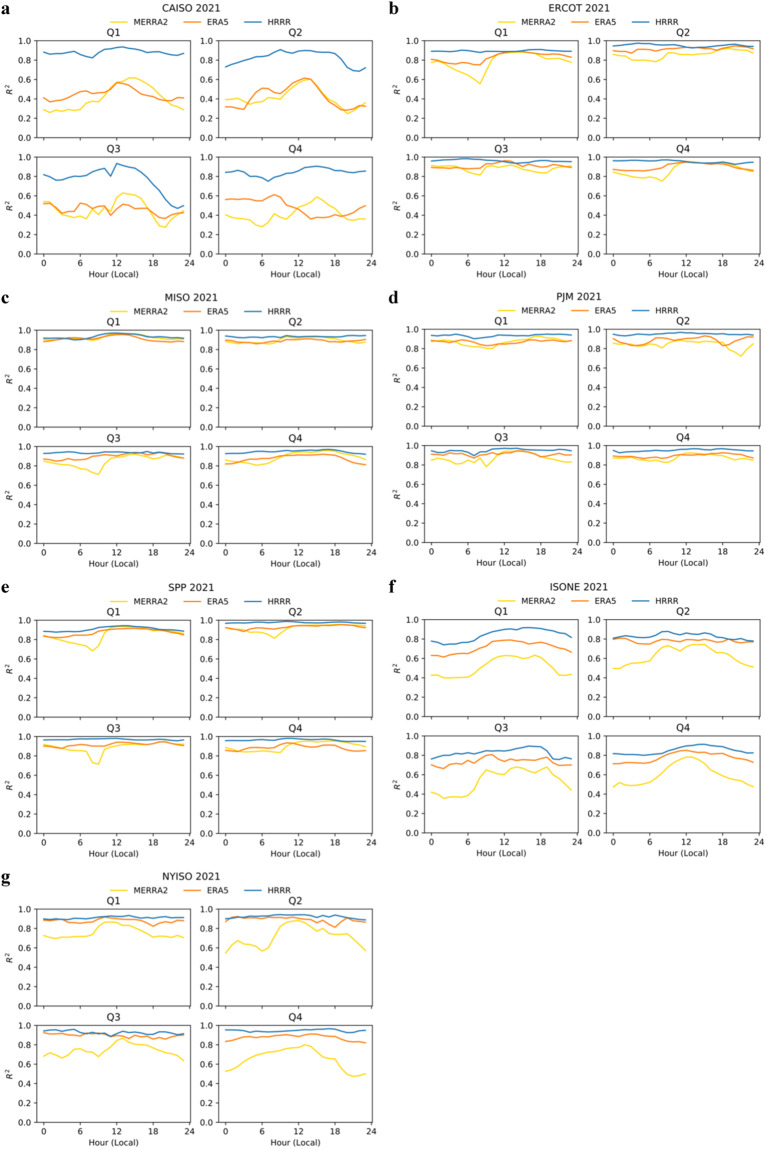
Seasonal and diurnal coefficient of determination (R^2^) for 2021 by model and ISO. ISOs include (**a**) CAISO, (**b**) ERCOT, (**c**) MISO, (**d**) PJM, (**e**) SPP, (**f**) ISO-NE, and (**g**) NYISO.

We also evaluate the models’ ability to represent variation in wind speed at each hour in the day through calculation of a coefficient of determination (COD). In this case, we separately assess each hour of the day for each quarter of the year in order to understand how well the models represent day-to-day variation at each particular hour in the day. Generally, the models accurately represent day-to-day variability in most regions and most hours of the day, Fig. 7. Specifically, the COD for all models is maintained above 0.8 in most regions and quarters, with HRRR consistently having a COD above 0.9. However, in certain cases the models cannot faithfully recreate day-to-day variance. In CAISO, for example, CODs for MERRA2 and ERA5 decline to ~0.5, varying by hour of the day. Also in CAISO, while CODs for HRRR are only slightly lower than in other regions, they drop noticeably in the late evening hours in Q2 and Q3. In ISO-NE, CODs were generally lower than elsewhere, though HRRR CODs declined only mildly compared to other regions. In NYISO, MERRA2 CODs fell below 0.8 during most nighttime hours. There are a number of hypotheses that could explain the decline in nighttime performance of the models, including related to model representation of the surface boundary layer, low level jets, and atmospheric stability. These hypothesis are discussed further in Davidson and Millstein^40^, which includes hourly and plant-level evaluation of these models in ERCOT.

### Summary of error and bias analysis

In terms of annual biases, ERA5 generally under predict wind generation while MERRA2 underpredicts principally in regions with complex terrain. HRRR generally has little bias, aside from California where it over predicts. More broadly, for each metric we have analyzed (i.e., annual bias, diurnal patterns, and day-to-day variability), all models have been most challenged by the same three regions: CAISO, NYISO, and ISO-NE. An obvious hypothesis for this challenge could be the higher complexity of terrain found in these regions versus the other regions, but further research is required to specifically tie model errors to specific causes. For the purpose of this data set, the comparison to recorded generation can be used to understand how one can appropriately use these data: each user can determine whether the biases and errors in different regions and at different times are sufficiently small for a specific application.

Though HRRR arguably had the best metrics across the time frames we analyzed, there are several reasons why researchers may want to use other models. MERRA2 and ERA5 are reanalysis models, and therefore offer global coverage and a consistent approach over time. HRRR, by contrast, is an operational meteorological model into which methodological improvements are continually introduced. Though these improvements likely improve model accuracy, they preclude analysis of long-term trends in output, as differences between years might be partially due to differences in the methods applied.

## Data Availability

Custom scripts were developed in R and python to process, manage, and clean the data. These scripts are available publicly at the repository https://github.com/AmosRAncell/PLUSWIND. Additionally, users may contact the corresponding author with questions about these scripts or about our source data.
